# Supplementary material for: Melanocortin 4 receptor-expressing neurons in the lateral stripe of the striatum regulate affect and motor control
Source: iScience. 2025 Apr 16;28(5):112456. doi: 10.1016/j.isci.2025.112456 (PMC12063154; doi:10.1016/j.isci.2025.112456)
Supplement: Document S1. Figure S1–S9 and Table S1 [file mmc1.pdf]

iScience, Volume *28*

Supplemental information

Melanocortin 4 receptor-expressing neurons in the lateral stripe of the striatum regulate affect and motor control

Johan Sköld, Gisela Paola Lazzarino, Myra Nett, Joost Wiskerke, and David Engblom

Supplemental information

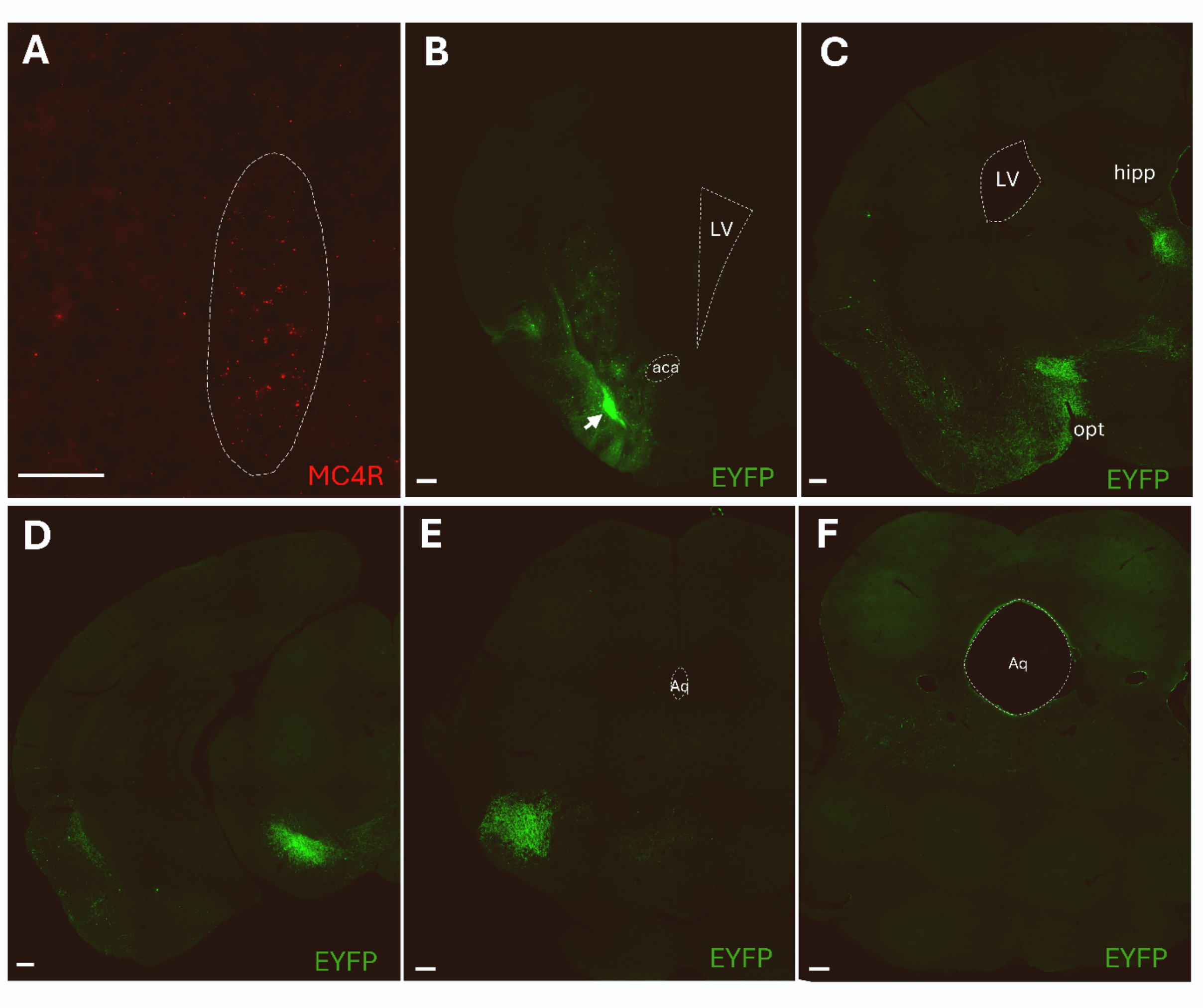

Figure S1. MC4R-neurons are present in the adult lateral stripe of the striatum and project to the substantia nigra pars reticulata, related to figure 1.

(A) Fluorescent in situ hybridization of a coronal section from an adult mouse showing ongoing MC4R-expression in the lateral stripe of the striatum (LSS). LSS marked with dashed line. Scale bar 100 µm. (B-F) Projection mapping from MC4R-LSS neurons. Injection of 100 nl AAV loaded with cre-dependent EYFP in the LSS. Selection of rostral-caudal sections stained with immunohistochemistry against EYFP. Expression of EYFP is seen at the injection site (B; arrow) and in projections to the lateral hypothalamus, epithalamus (C) and midbrain. Strong immunostaining can be seen in the substantia nigra pars reticulata (D-F). Scale bars 200 µm. Lateral ventricle (LV), anterior commissure (aca) and cerebral aqueduct (aq) marked with dashed lines. hipp: Hippocampus, opt: optic tract.

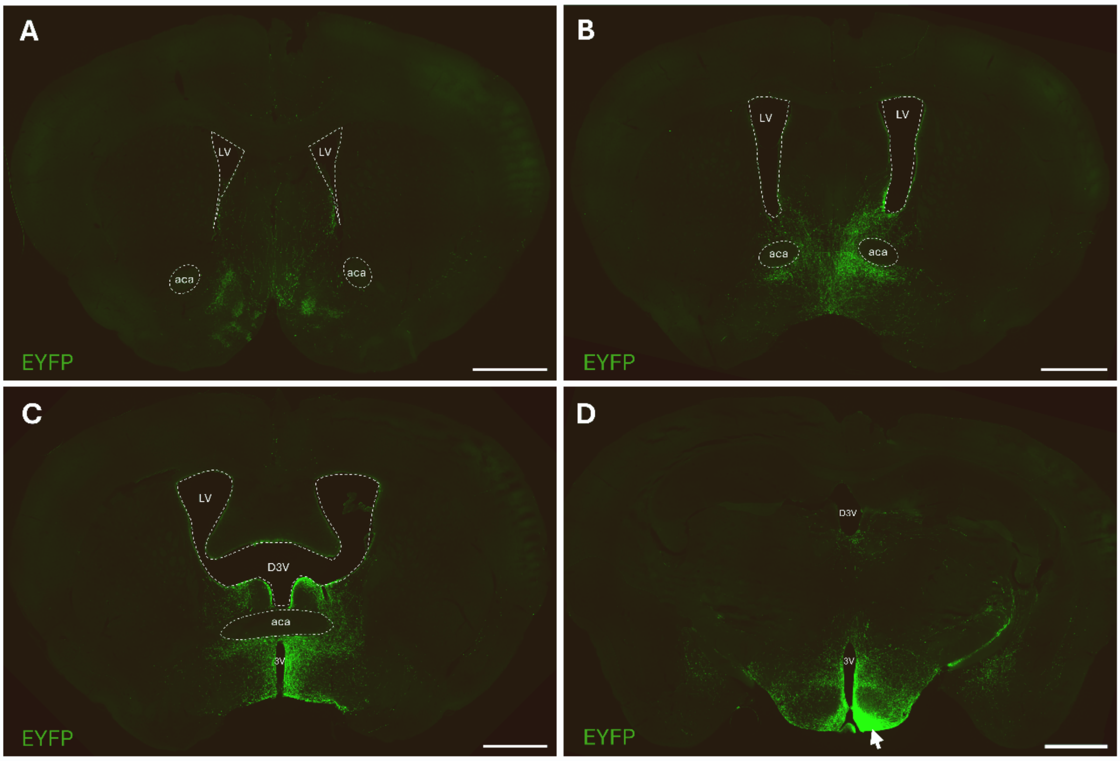

Figure S2. No projections from arcuate nucleus POMC-neurons were found in the lateral stripe of the striatum, related to figure 1.

(A-D) Rostral-caudal images from a POMC-cre mouse injected with cre-dependent EYFP in the arcuate nucleus. Projections are seen in the medial nucleus accumbens shell and other areas, but not in the lateral stripe of the striatum (LSS) or the dorsal striatum. Injection site shown in D (arrow). Scale bars 200 µm. Lateral ventricle (LV), anterior commissure (aca) and marked with dashed lines. 3V: third ventricle, D3V: dorsal third ventricle.

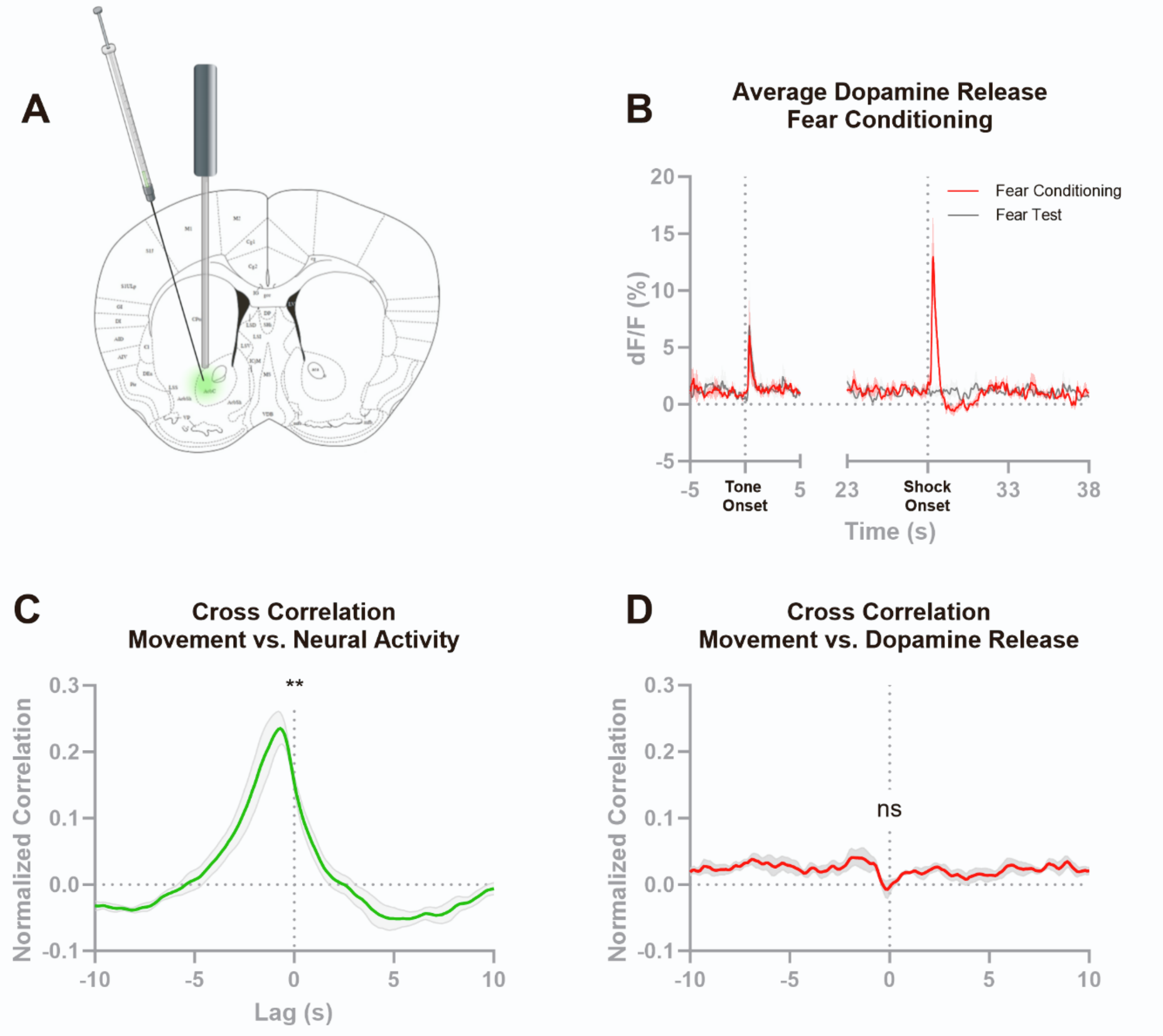

Figure S3. Dopamine is released in the nucleus accumbens core by aversive foot shocks and their paired cues, related to figure 2 and 3.

(A) Schematic showing site of injection and fiber placement in the nucleus accumbens core (NAcC). (B) Dopamine release measured with dLight1.2 from the NAcC during fear conditioning and fear test the following day. Dopamine is released in response to aversive foot shocks and their predictive tone (mean from 6 trials per mouse, n = 4 mice). (C) Cross-correlation of neural activity of unspecified neurons of the NAcC, measured with constitutive GCaMP6s, and body movements of the mouse during fear test. A clear correlation is seen between neural activity and body movements. R-values at lag 0 are significantly different from 0 ($t_2 = 12.72$, $p < 0.01$, n = 3, one sample t-test). (D) Cross-correlation of dopamine release in the NAcC and body movements of the mice during fear test show that dopamine release is uncorrelated to movement of the animal R-values at lag 0 are not significantly different from 0 ($t_3 = 0.34$, ns, n = 4, one sample t-test). **$p < 0.01$. Data expressed as mean±SEM.

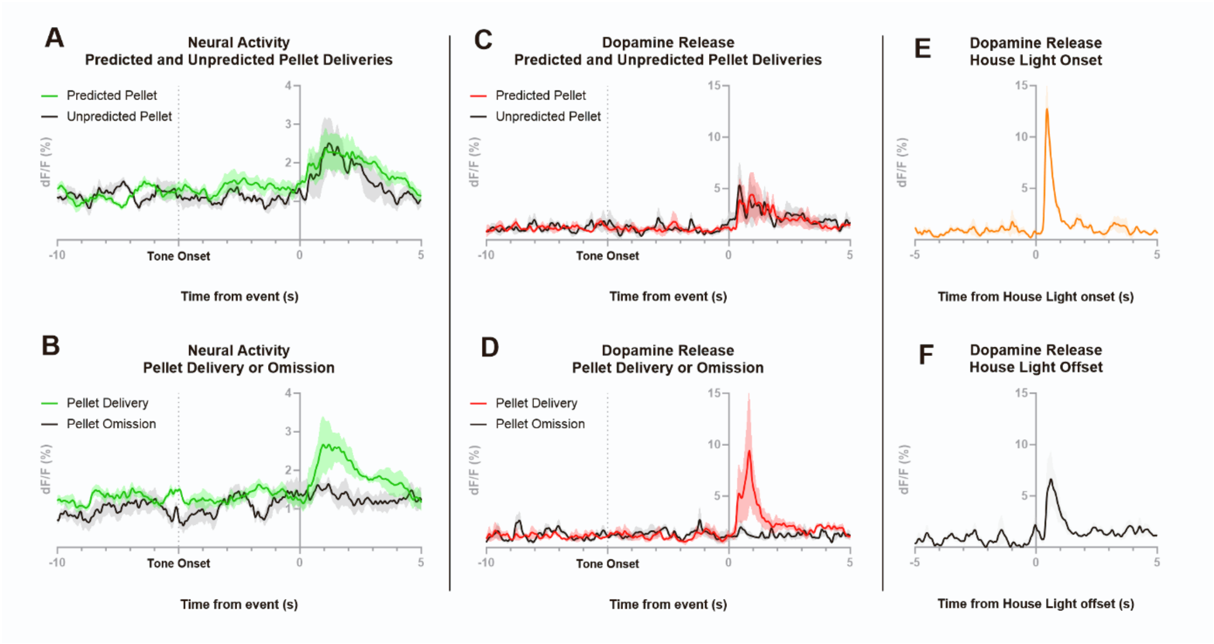

Figure S4. No reward prediction error encoding by MC4R-expressing neurons or dopamine in the lateral stripe of the striatum, related to figure 4.

(A) The neural response, measured with GCaMP8m, is similar after predicted and unpredicted pellets, suggesting there is no positive reward prediction error encoded by the MC4R-lateral stripe of the striatum (LSS) neurons (mean from 20 predicted pellets and 10 unpredicted pellets per mouse, n = 6 mice) (B) The neural activity is not decreased by omission of the predicted reward, suggesting there is no negative reward prediction error encoded by MC4R-LSS neurons (mean from 20 predicted pellets and 10 omissions per mouse, n = 6 mice). (C-D) Same as in (A-B), but for dLight1.2 measurements of dopamine release. (C) Similar dopamine release was seen in response to predicted and unpredicted pellet deliveries, suggesting there is no positive reward prediction error encoded by dopamine release in the LSS. (mean from 20 predicted pellets and 10 unpredicted pellets per mouse, n = 5 mice) (D) The dopamine release is not decreased by omission of the predicted reward, suggesting there is no negative reward prediction error encoded by dopamine release in the LSS (mean from 20 predicted pellets and 10 omissions per mouse, n = 5 mice). (E) Dopamine is released in the LSS in response to house light turning on (mean from 5 training sessions, n = 5 mice). (F) Dopamine is released in the LSS in response to house light turning off (mean from 5 training sessions, n = 5 mice). Data expressed as mean±SEM.

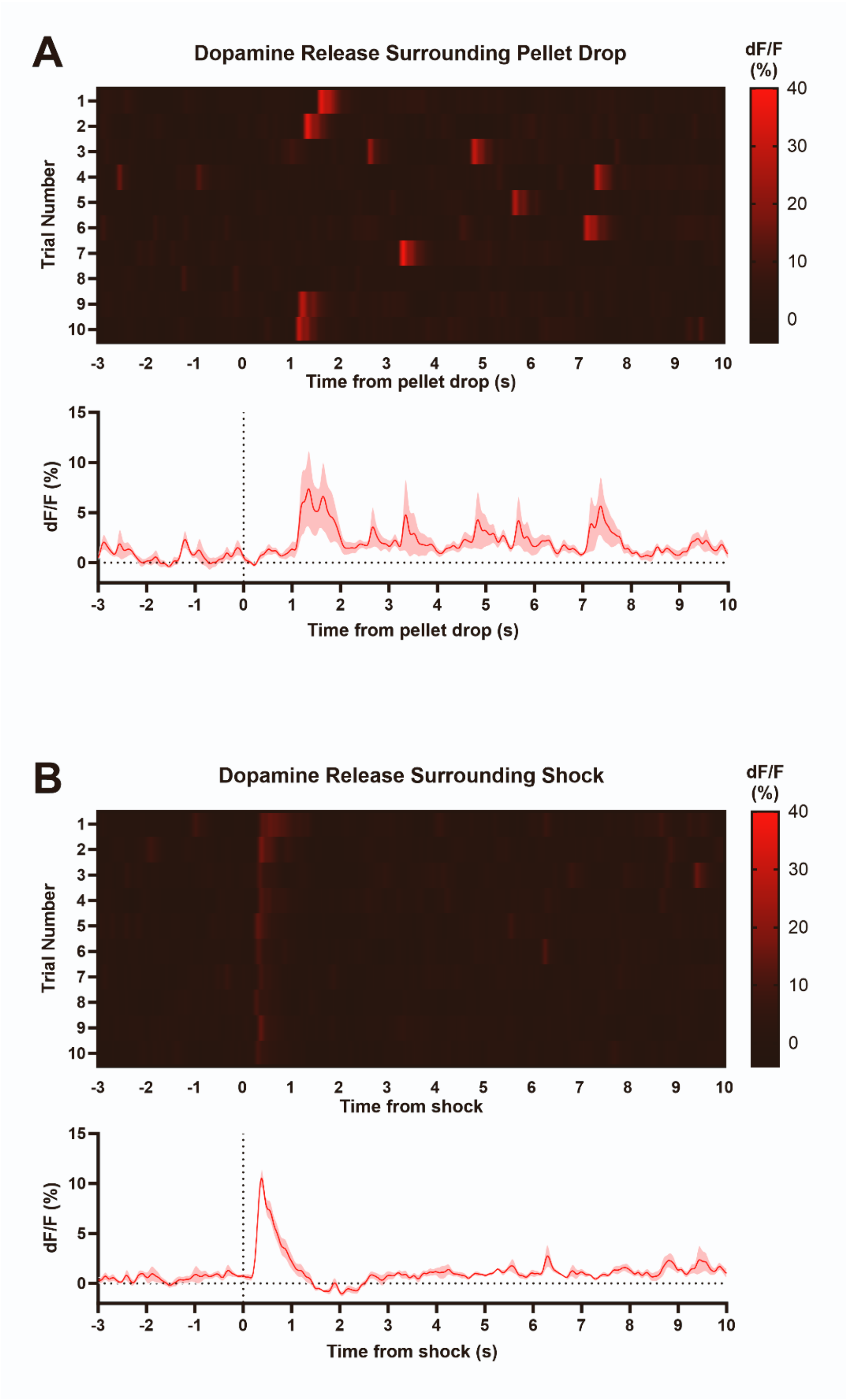

Figure S5. Different temporal dynamics of dopamine release to sugar pellet consumption and foot shock, related to figure 4.

Representative heatmaps and mean±SEM dF/F traces of dLight1.2 recordings from the LSS from a single animal during (A) 10 sugar pellet deliveries and (B) 10 foot shock events illustrating the different temporal patterns of dopamine release in response to the different trial types. While sugar pellet consumption triggers large dopamine transients, the spread in time of the events flattens the mean trace.

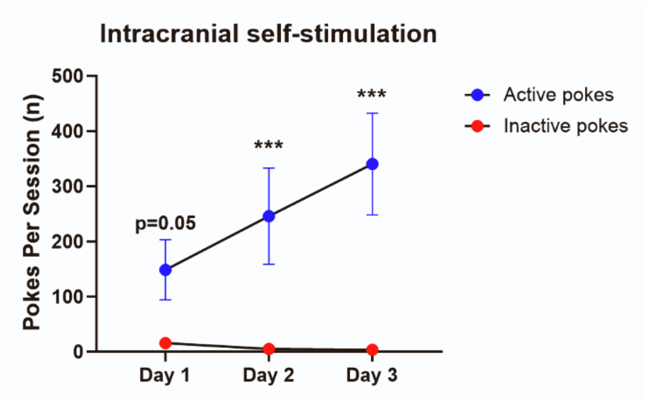

Figure S6. ChR2-expressing mice selectively nose-poke for unilateral optogenetic activation of MC4R-LSS neurons, related to figure 5.

Mice showed a significant preference for nose-poking in the port that deliver 2-second unilateral stimulation of MC4R-LSS neurons (active pokes) compared to the port that did not deliver stimulation (inactive pokes) in an optogenetic intracranial self-stimulation paradigm ($n = 9$, repeated-measures ANOVA followed by Šídák's multiple comparisons test). ***$p < 0.001$.

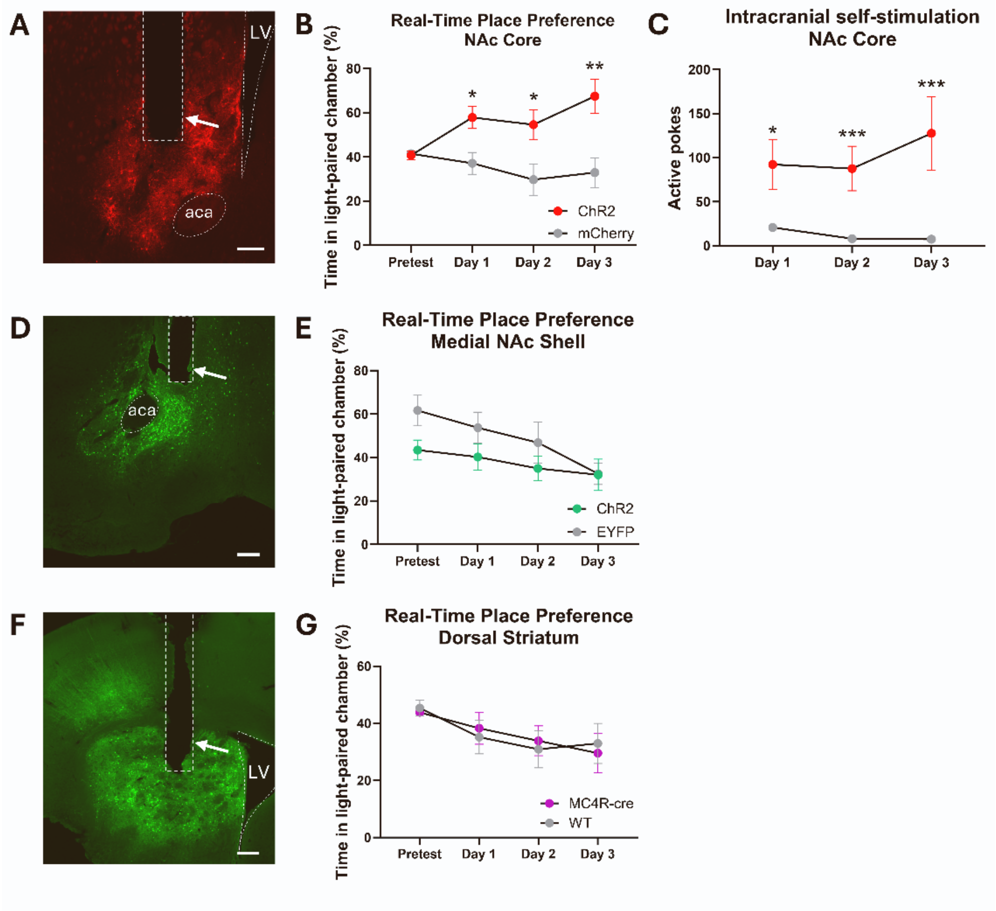

Figure S7. Activating MC4R-neurons in the nucleus accumbens core, but not in the medial nucleus accumbens shell or in the dorsal striatum, is rewarding, related to figure 5.

(A) Representative picture showing viral expression (pAAV5-EF1a-double floxed-hChR2(H134R)-mCherry-WPRE-HGHpA) and fiber placement of MC4R-cre animals injected in nucleus accumbens (NAc) core. Lateral ventricle (LV) and anterior commissure (aca) marked with dashed lines. Fiber tract marked with dashed lines and pointed out with an arrow. (B) Mice with opsin expression (ChR2) in nucleus accumbens core neurons prefer a chamber paired with light stimulation, contrary to EYFP controls (F_{Opsin} (1, 20) = 9.20, p < 0. 01, $F_{TimexOpsin}$ (3, 60) = 7.17, p < 0.001, n = 12 ChR2, n = 10 mCherry, repeated-measures ANOVA followed by Šídák's multiple comparisons test, ChR2 vs. mCherry). (C) Mice self-stimulate to optogenetically activate MC4R-neurons in the nucleus accumbens core (F_{Opsin} (1, 26) = 22.06, p < 0.001; $F_{TimexOpsin}$ (2, 52) = 5.36, p < 0.01, n = 16 ChR2, n = 12 mCherry controls, repeated-measures ANOVA followed by Šídák's multiple comparisons test, ChR2 vs. mCherry). (D) Representative picture showing viral expression (pAAV5-EF1a-doublefloxed-hChR2(H134R)-EYFP-WPRE-HGHpA) and fiber placement of MC4R-cre animals injected in medial nucleus accumbens shell. Anterior commissure (aca) marked with dashed lines. Fiber tract marked with dashed lines and pointed out with an arrow. (E) No effect was seen in a real-time place preference paradigm in response to optogenetic activation of MC4R-neurons in the medial nucleus accumbens shell (F_{Opsin} (1, 12) = 3.09, ns; $F_{TimexOpsin}$ (3, 36) = 0.88, ns, n = 8 ChR2, 6 EYFP controls, repeated-measures ANOVA followed by Šídák's multiple comparisons test, ChR2 vs. EYFP). (F) Representative picture showing viral expression (pAAV5-EF1a-doublefloxed-hChR2(H134R)-EYFP-WPRE-HGHpA) and fiber placement of MC4R-cre animals injected in dorsal striatum. Lateral ventricle (LV) marked with dashed lines. Fiber tract marked with dashed lines and pointed out with an arrow. (G) No effect was seen in a real-time place preference paradigm in response to optogenetic activation of MC4R-neurons in the dorsal striatum (F_{Strain} (1, 13) = 0.002, ns; $F_{TimexStrain}$ (3, 39) = 0.35, ns, n = 8 MC4R-cre, 7 wildtype (WT) littermate controls, repeated-measures ANOVA followed by Šídák's multiple comparisons test, MC4R-cre vs.

WT). Scale bars 200 µm. LV: Lateral ventricle, aca: anterior commissure. *$p < 0.05$; **$p < 0.01$; *** $p < 0.001$.

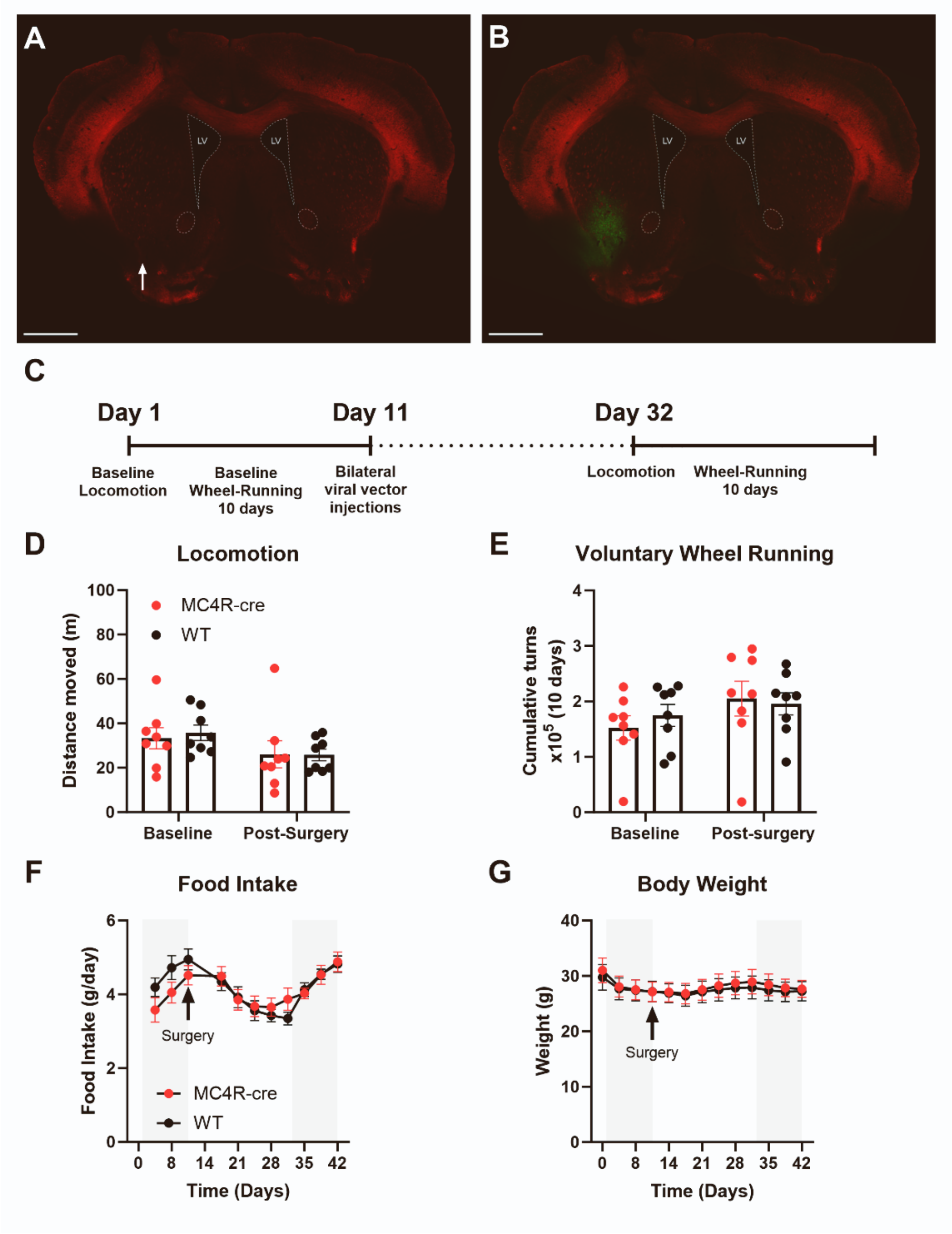

Figure S8. Ablation of MC4R-LSS neurons does not affect locomotor or appetitive behaviors, related to figure 5.

(A-B) Validation of caspase3-ablation of MC4R-LSS-neurons. Representative picture of MC4R-cre-tdTomato mouse injected with cre-dependent caspase3 and constitutive EYFP in the lateral stripe of the striatum (LSS). Scale bars 1 mm. (A) No tdTomato-expressing MC4R neurons can be seen in the injected side (arrow) while the LSS is clearly visible on the control side. (B) Overlap with EYFP expression showing the spread of viral vector injection. Anterior commissures and lateral ventricles (LV) marked with dashed lines. (C) Timeline of experiment shown in (D-E). (D) Open field locomotion is not affected by ablation of MC4R-LSS neurons. (E) Ablation of MC4R-LSS neurons does not affect voluntary wheel-running behavior. (F) Food intake was measured every 3-4 days during the experiment. Data expressed as grams/day. No difference was seen between mice with ablated MC4R-LSS neurons compared to littermate controls. (G) No difference in body weight seen before or

after ablation of MC4R-LSS neurons compared to wildtype littermates. Shaded areas in (F-G) indicate time in wheel-running boxes. n=8 per group.

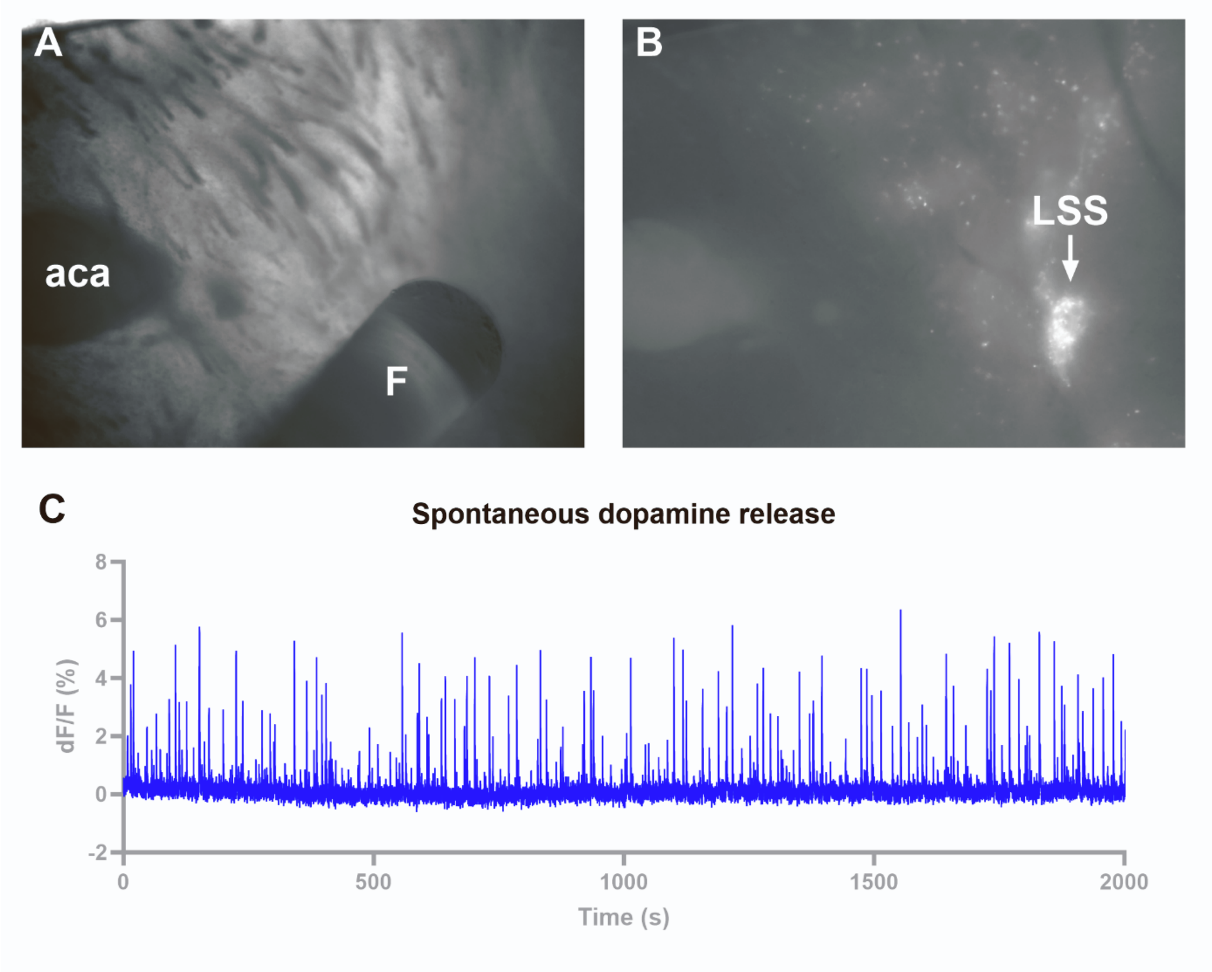

Figure S9. Spontaneous dopamine release can be measured from a coronal slice using fiber photometry, related to figure 6.

(A) Picture showing fiber placement over the lateral stripe of the striatum (LSS) in a coronal slice of the striatum. aca: a commissure, F: optic fiber. (B) Expression of rsChrmine in the LSS in the same coronal slice as in (A). (C) Example graph showing spontaneous dopamine peaks in a coronal slice proving that dopamine release occurs and can be measured under the experimental conditions.

Figure	Statistical test	Comparison	n	df	t/F	p	Figure display
2K	Paired t-test		7	6	0.5605	0.5954	ns
2L	Paired t-test		11	10	7.172	<0.0001	****
3B	One-sample t-test		6	5	9.623	0.0002	**
3D	One-sample t-test		7	6	0.8643	0.4206	ns
3F	One-sample t-test		10	9	2.630	0.0274	*
4E	One-sample t-test	Pellet	6	5	8.50	0.0004	***
4E	One-sample t-test	Shock	6	5	16.7	<0.0001	***
4G	One-sample t-test	Shock	5	4	4.86	0.0083	**
4G	Paired t-test		5	4	5.713	0.0046	#
4H	One-sample t-test	Pellet	5	4	5.04	0.0073	**
4H	One-sample t-test	Shock	5	4	3.19	0.0331	*
4H	Paired t-test		5	4	3.024	0.0390	#
5D	2-way Anova, Šídák's multiple comparisons test	Day 1	7+9	9.328	2.939	0.047	*
5D	2-way Anova, Šídák's multiple comparisons test	Day 2	7+9	13.87	5.334	0.0003	***
5D	2-way Anova, Šídák's multiple comparisons test	Day 3	7+9	12.01	4.369	0.0027	**
5E	2-way Anova, Šídák's multiple comparisons test	Day 1	7+9	56	3.024	0.015	*
5E	2-way Anova, Šídák's multiple comparisons test	Day 2	7+9	56	3.022	0.015	*
5E	2-way Anova, Šídák's multiple comparisons test	Day 3	7+9	56	3.264	0.0075	**
5F	2-way Anova, Šídák's multiple comparisons test	5-10	7+9	42	2.957	0.0152	*
6D	2-way Anova, Šídák's multiple comparisons test	0 vs 5	6+6	36	3.173	0.0304	*
6D	2-way Anova, Šídák's multiple comparisons test	0 vs 10	6+6	36	5.534	<0.0001	***
6D	2-way Anova, Šídák's multiple comparisons test	0 vs 15	6+6	36	6.258	<0.0001	***
6F	2-way Anova, Šídák's multiple comparisons test	2 vs 20	6+6	36	4.17	0.0018	**
6F	2-way Anova, Šídák's multiple comparisons test	2 vs 40	6+6	36	5.725	<0.0001	**
6F	2-way Anova, Šídák's multiple comparisons test	5 vs 40	6+6	36	4.17	0.0018	**
6F	2-way Anova, Šídák's multiple comparisons test	10 vs 20	6+6	36	3.631	0.0087	**
6F	2-way Anova, Šídák's multiple comparisons test	10 vs 40	6+6	36	5.187	<0.0001	**

S3C	One-sample t-test		3	2	12.72	0.0061	**
S3D	One-sample t-test		4	3	0.3398	0-7564	ns
S6	2-way Anova, Šídák's multiple comparisons test	Day 2	9+9	12.74	5.701	<0.0001	***
S6	2-way Anova, Šídák's multiple comparisons test	Day 3	9+9	10.54	4.929	0.0005	***
S7B	2-way Anova, Šídák's multiple comparisons test	Day 1	12+11	80	2.549	0.0498	*
S7B	2-way Anova, Šídák's multiple comparisons test	Day 2	12+11	80	3.061	0.012	*
S7B	2-way Anova, Šídák's multiple comparisons test	Day 3	12+11	80	4.251	0.0002	**

S7C	2-way Anova, Šídák's multiple comparisons test	Day 1	16+12	78	2.584	0.0345	*
S7C	2-way Anova, Šídák's multiple comparisons test	Day 2	16+12	78	4.593	<0.0001	***
S7C	2-way Anova, Šídák's multiple comparisons test	Day 3	16+12	78	5.182	<0.0001	***

Table S1. Statistical summary, related to STAR methods.
